# Prevalence, awareness, treatment, control, and risk factors of hypertension among adults: a cross-sectional study in Iran

**DOI:** 10.4178/epih.e2018020

**Published:** 2018-05-18

**Authors:** Maryam Eghbali, Alireza Khosravi, Awat Feizi, Asieh Mansouri, Behzad Mahaki, Nizal Sarrafzadegan

**Affiliations:** 1 Nursing and Midwifery Care Research Center, School of Nursing and Midwifery, Isfahan University of Medical Sciences, Isfahan, Iran; 2 Interventional Cardiology Research Center, Isfahan Cardiovascular Research Institute, Isfahan University of Medical Sciences, Isfahan, Iran; 3 Department of Biostatistics and Epidemiology, School of Public Health, Isfahan University of Medical Sciences, Isfahan, Iran; 4 Hypertension Research Center, Isfahan Cardiovascular Research Institute, Isfahan University of Medical Sciences, Isfahan, Iran; 5 Department of Biostatistics, School of Public Health, Kermanshah University of Medical Sciences, Kermanshah, Iran; 6 Isfahan Cardiovascular Research Center, Isfahan Cardiovascular Research Institute, Isfahan University of Medical Sciences, Isfahan, Iran

**Keywords:** Blood pressure, Epidemiology, Hypertension, Chronic diseases, Risk factors, Iran

## Abstract

**OBJECTIVES:**

Hypertension (HTN) is an important risk factor for cardiovascular disease. Considering the importance of this disease for public health, this study was designed in order to determine the prevalence, awareness, treatment, control, and risk factors of HTN in the Iranian adult population.

**METHODS:**

This cross-sectional study was conducted among 2,107 residents of Isfahan, Iran. Samples were selected through multi-stage random cluster sampling in 2015-2016. The outcome variable was HTN, determined by measuring blood pressure in the right arm via a digital arm blood pressure monitor. Awareness, treatment, and control of HTN were assessed by a validated and reliable researcher-developed questionnaire. Other demographic and clinical variables were assessed via a demographic questionnaire.

**RESULTS:**

The overall prevalence of HTN was 17.3% (18.9 and 15.5% in men and women, respectively). The prevalence of HTN increased in both genders with age. The prevalence of awareness of HTN among people with HTN was 69.2%, of whom 92.4 and 59.9% were taking medication for HTN and had controlled HTN, respectively. Logistic regression identified age, body mass index, having diabetes and hyperlipidemia, and a positive family history of HTN as determinants of awareness of HTN.

**CONCLUSIONS:**

The results showed that HTN was highly prevalent in the community, especially in men and in middle-aged and older adults. Approximately 30.8% of patients were unaware of their disease, and there was less awareness among younger adults. Despite the high frequency of taking medication to treat HTN, it was uncontrolled in more than 40.1% of patients. Health policy-makers should therefore consider appropriate preventive and therapeutic strategies for these high-risk groups.

## INTRODUCTION

Hypertension (HTN) is a major independent and progressive risk factor for cardiovascular disease (CVD), with significant economic and health complications around the world [1]. In 2010, it was found to be 1 of the 3 major risk factors contributing to the global disease burden [2]. According to the results of a study in Tehran, HTN was the most important cardiac risk factor for ischemic stroke [3]. In China, of the 230 million people with CVD, about 200 million also have HTN [4]. In general, the prevalence of HTN among adults is higher in low- and middle-income countries (about 40.0%) than in high-income countries (35.0%). It also affects more people in these countries because of their high population density. In addition, because of the weakness of the health systems in these countries, the number of undiagnosed, untreated, and uncontrolled cases is also higher [5]. The highest prevalence of HTN was found to be 46.0% among adults in Africa, in contrast to 35.0% among American adults [6] and roughly 23.0% among Canadian adults [7]. According to the results of a longitudinal study with a 7-year follow-up in Isfahan, cardiovascular events were more common among urban dwellers than among rural residents, and HTN was the strongest predictor of cardiovascular events in urban men and women [8].

In the last decade, the rapid social and economic changes in the Eastern Mediterranean and Middle Eastern countries have led to a surge of many cardiovascular risk factors, including HTN. According to a report by the United Nations, the average prevalence of HTN is 26.0% in Eastern Mediterranean countries [9].

Despite the importance of HTN for health, many patients with HTN are not aware of their disease. According to a previous study [10], the prevalence of awareness of HTN among urban residents with HTN in high-, low-, low–middle- and upper–middle-income countries was 48.3, 48.4, 49.3, and 52.1%, respectively [10]. In an Iranian population with HTN, the corresponding rate was 40.6% [11]. Malekzadeh et al. [12] estimated the prevalence of HTN awareness to be 46.2% in their Golestan cohort study.

Most people with HTN consider themselves to be healthy and do not feel the need for treatment until they experience complications. They only become motivated and inclined to seek care and change their lifestyle when their disease has progressed and they have developed serious complications [13]. Research has shown that the early detection and treatment of HTN and its risk factors, as well as public health policies to reduce behavioral risk factors, have led to a gradual reduction in mortality caused by heart disease and stroke in high-income countries in the past 3 decades [5]. The aims of our study were to determine the prevalence of HTN and its risk factors among adults and to determine the prevalence of awareness, pharmacological treatment, and control of HTN among adults with HTN.

## MATERIALS AND METHODS

### Study design and participants

This cross-sectional study was conducted among adults over age 18 residing in Isfahan, one of the major metropolises of Iran, from August 2015 to March 2016. The sample size was 2,107. The subjects were selected through multi-stage random cluster sampling and the households were randomly selected in proportion to the population covered by the relevant health centers. More details about the design and sampling have been presented elsewhere [14].

### Data collection, variables, and tools

The data collected by the questionnaire included demographic characteristics and clinical information. A validated and reliable researcher-developed questionnaire was utilized in this study [14]. At the health center, the candidates were first asked to sign a written consent form and then, while seated in a chair, answered questions about their demographic details, including age, gender, years of education, marital status, economic status, occupation, and history of diabetes and elevated blood lipids. They then relaxed in a quiet environment for 5 minutes. The World Health Organization (WHO) standards for taking blood pressure were met [15], and blood pressure in the right arm in the seated position was measured and recorded 3 times at 1-minute intervals. The mean of the second and third measurements was considered as the subject’s blood pressure. If the mean systolic blood pressure (SBP) was ≥ 140 mmHg and/or the mean diastolic blood pressure (DBP) was ≥ 90 mmHg, or if the subject reported having been diagnosed with HTN and taking antihypertensive medications, he or she was considered a case of HTN. If the subject reported having been diagnosed with HTN by a health professional or taking medication for high blood pressure, he or she was considered to be a case of HTN who was aware of the disease [16].

The subjects’ height was measured in centimeters in a standing position using a non-elastic measuring tape mounted on the wall and calibrated with a metal measuring tape. Their weight was measured in kilograms without shoes and while wearing light-weight clothing using a digital scales (Soehnle, Nassau, Germany).

The prevalence of awareness, pharmacological treatment, and control of HTN was determined according to definitions presented by Gee et al. [16]. According to this reference, “proportion of adults with HTN who report either having been diagnosed with HTN by a health professional or who report taking medication for high blood pressure, proportion of adults with HTN who report taking medication for high blood pressure and proportion of adults with HTN who both (1) report taking medication for high blood pressure and (2) have SBP <140 mmHg and DBP <90 mmHg” were used as criteria for the prevalence of awareness, pharmacological treatment, and control of HTN, respectively [16].

The data collection tools used in this study included an digital arm blood pressure monitor (Microlife, Widnau, Switzerland), which was compared to a mercury sphygmomanometer and was verified several times on 1-3 individuals.

Since blood pressure is a very sensitive variable with physiological fluctuations over time [17,18], it is essential to notice its intrasubject, intra- and inter-observer variation during measurements. These forms of variation were minimized in our study in the following ways. To address intra-subject variation, as mentioned above, we applied the WHO standards for taking blood pressure. These standards ensure that a patient’s blood pressure is measured in a stable condition. According to the WHO STEPwise approach to surveillance protocol, each patient’s blood pressure was measured by a properly calibrated digital automatic blood pressure monitor [19]. We were not significantly concerned about intra- and inter-observer variation for 3 reasons. First, the observers were thoroughly trained and completely familiar with working with this tool and ensuring its correct operation. Second, the value of blood pressure for each patient was considered as the numbers displayed on the tool’s monitor screen, instead of the traditional method that uses the observer’s hearing, meaning that our observations were not affected by random errors in the observer’s hearing. Third, the tool used for measuring blood pressure was the same for all participants.

The other instruments included a non-elastic measuring tape calibrated with a metal tape and a digital scale that was calibrated every day with a control 5 kg weight. The researcher trained the interviewers on how to work with the measuring tools and issued official letters of certification for them after ensuring that they had developed the necessary skills.

The inclusion criteria were residing in Isfahan and being age 18 and over. The exclusion criteria were fasting or engaging in a weight gain or weight loss diet at the time of the study; having chronic diseases, including kidney failure and cancer, as per the participant’s report; and pregnancy for women participants.

### Ethical considerations

The study was ethically approved under the code Ir.mui.rec.2016. 3.790. The study objectives were explained to each of the subjects and written consent was obtained from them. The participants’ personal information remained confidential.

### Statistical analysis

Data were analyzed in SPSS version 19 (IBM Corp., Armonk, NY, USA). The numerical variables were reported as mean and standard deviation (SD) and the non-numerical variables as number and percentage. The independent *t*-test was used to compare the numerical variables in the men and women groups. The chisquare and binomial tests were used to compare non-numerical values and prevalence between the men and women subjects. Multiple logistic regression was used to identify determinants of awareness of HTN.

## RESULTS

This study was conducted among 2,107 adult residents of Isfahan, including 1,017 women (48.3%) and 1,090 men (51.7%) aged 18 to 84. The mean± SD of age was 39.53± 15.74 years. The demographic and clinical characteristics of participants are presented in Table 1. As shown, our participants were on average middle-aged, overweight, with normal SBP and DBP, married, homemakers (among women), and with moderate education and economic status. The prevalence of diabetes and hyperlipidemia was considerable in our participants. When we compared these characteristics between men and women, we observed significant differences in body mass index (BMI), SBP, DBP, education, marital status, and occupation. According to these comparisons, women had a significantly higher BMI, lower SBP, and lower DBP, and were more likely to be unemployed and single than men.

A total of 364 participants (17.3%) had HTN. The number (percentage) of HTN cases among women and men was 158 (15.5%) and 206 (18.9%), respectively. We compared the prevalence of certain cardiovascular risk factors between people with and without HTN. The results are presented in Table 2. As shown, people with HTN were significantly older and had a higher BMI. The prevalence of diabetes, hyperlipidemia, and a positive family history of HTN, as well as men gender, was significantly higher in people with HTN than in those without HTN. We did not observe significant differences in smoking and stress between the 2 groups.

**Figure 1.**
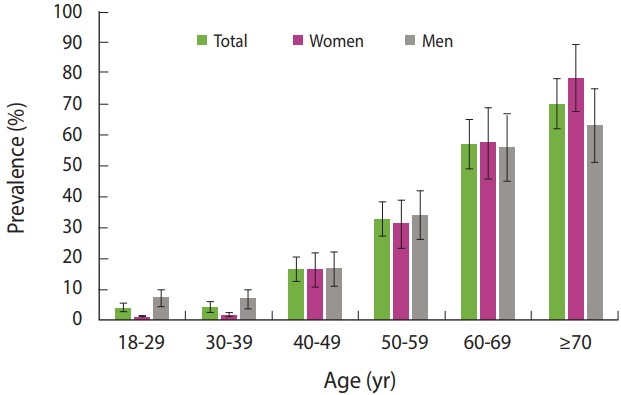
Age variations in the prevalence of hypertension among women and men in Isfahan, Iran, 2016. The error bars indicate 95% confidence intervals of prevalence.

We compared HTN prevalence between age and gender groups. The results are presented in Table 3. The prevalence of HTN significantly increased with age (p< 0.05). This trend was observed in both men and women (p< 0.05). Figure 1 shows variations in the prevalence of HTN in total and among the age groups of women and men. The error bars indicate the 95% confidence intervals (CIs) of prevalence.

We assessed the prevalence of awareness of HTN in total and by age. We then determined the prevalence of pharmacological treatment for HTN and having controlled HTN among people with HTN who were aware of their disease. The results are presented in Table 4. The prevalence of awareness of HTN among people with HTN was 69.2, of whom 92.4 and 59.9% were taking medication for HTN and had controlled HTN, respectively. We observed a significant incremental trend for higher awareness of HTN and pharmacological treatment of HTN according to age, but no such trend was found for controlled HTN.

We assessed the relationship between certain cardiovascular risk factors and the awareness of HTN via multiple logistic regression. The results are presented in Table 5. We found that the odds of awareness of HTN significantly increased with age, BMI, having diabetes and hyperlipidemia, and a positive family history of HTN.

## DISCUSSION

The woman participants in this study had a significantly higher BMI than the man participants. In another study in Iran, the prevalence of obesity was higher in women (40.0%) than in men (25.4%). The researchers reported that being a homemaker was one of the potential causes of women obesity [20]. In the study of Garawi et al. [21] the prevalence of overweight was higher in women than in men, and they considered the lack of regular physical activity and non-adherence to a healthy diet to be contributing factors.

In the present study, years of education and the employment rate were higher in men than in women. The SBP and DBP were also significantly higher in men. The mean± SD of SBP and DBP were 117.77± 15.81 and 72.21± 10.20 mmHg in the present study, respectively. In the study of Ebrahimi et al. [22] in 30 provinces of Iran, the mean SBP and DBP were 116.24 and 74.58 mmHg, respectively.

In this study, the prevalence of cardiovascular risk factors was higher in individuals with HTN than in the general population. Other studies have found the prevalence of metabolic syndrome to be higher in individuals with HTN than in the general population. Conversely, HTN was present in 80.0% of individuals with metabolic syndrome [23].

The prevalence of HTN was observed to be 17.3% in Isfahan in this study. In the study of Ebrahimi et al. [22] of 30,000 people aged 15 to 64 across Iran, the prevalence of HTN was 17.4%. In a study of Isfahan Healthy Heart Program (IHHP), the overall prevalence of HTN among rural and urban adults in Isfahan, Najafabad, and Arak was 18.9% [24]. In a study conducted in 2015 in East Azerbaijan Province in Iran that included 2,818 people, the prevalence of HTN was 22.6% [25]. Differences in the prevalence of HTN in different regions of Iran can be attributed to differences in age groups, education, culture, economy, geography, lifestyle, and nutritional habits [23,26], as well as the method of sampling.

According to the results of this study, when adopting strategies for the prevention and control of HTN, gender and age differences should be taken into account, as in this study, the prevalence of HTN increased with age, and the highest prevalence was observed in those over 70 and the lowest in the 18-29 age group. Additionally, in both men and women, the prevalence of HTN was higher in the older age groups. One of the reasons for this finding is that changes take place in the vascular walls as part of the physiological factors associated with age that affect blood pressure. The prevalence of HTN risk factors, such as diabetes, obesity, and high cholesterol, also increases with age. A direct relationship was found between age and HTN in both genders in a systematic review conducted from 1980 to 2012 in Iran [27]. In a study by Choi et al. [28] of 27,887 people over age 30 using the data from the fifth (2010 to 2012) and sixth (2013 to 2014) Korean National Health and Nutrition Examination Surveys, the prevalence of HTN increased in both genders with age.

In a study by Ghorbani et al. [11] in Iran, the prevalence of HTN was strongly and significantly associated with age, such that 42.5% of the people in the 50-69 age group had HTN. According to a systematic review, in 27 provinces of Iran, the overall prevalence of HTN was about 23.0% in the 30-55 age group and about 50.0% in the over-55 age group [29].

In this study, the prevalence of HTN was higher in men, in contrast to the study by Tabrizi et al. [25] that reported the prevalence of HTN to be higher in women. The reason for this might be that in the study of Tabrizi et al. [25], the subjects were not selected according to both their gender and age ratios, while the present study accounted for both these ratios. In a cross-sectional study by Chow et al. [10] of 142,042 people in the 35-70 age group in 17 countries from 5 continents, the prevalence of HTN was higher in men.

In this study, the prevalence of HTN was higher in men in the 18-29 and 30-39 age groups. That is, men younger than 40 (6.5%) were more likely to have HTN than women younger than 40 (0.6%), so more attention should be paid to this particular group. The prevalence of HTN did not differ significantly by gender in the 40-and-over age group and was as high in women (45.7%) as in men (42.4%). These findings indicate a significant increase in the prevalence of HTN in women near the age of menopause and older. In a study by Zhou et al. [30] of 6,324 women aged 35 and over, although menopause was identified as an independent risk factor for HTN, other risk factors for HTN, such as obesity, abdominal obesity, diabetes, and elevated triglycerides, also increased in women with menopause.

HTN does not have any specific signs or symptoms. The absence of symptoms means that the disorder is ignored by many people, and this negligence can have very dangerous health complications, which is why HTN is known as the silent killer. According to the results of this study, 69.2% of people were aware of their HTN and about 30.8% were unaware. In a study in urban and rural areas of Semnan province in Iran, 40.6% of people were aware of having HTN, and in another study, 45.8% of people were aware of having HTN [11]. The reason for the disparity between those findings with the findings of the present study is that the present study was carried out in urban areas of Isfahan, and those living in villages were not included in the sampling.

According to large-scale epidemiological studies, the awareness of people with HTN of their disease is not satisfactory. Moreover, people with HTN do not take enough medication, or the correct medication, even after becoming aware of having HTN [31]. In this study, the prevalence of awareness of HTN was significantly higher in older age groups, which can be attributed to the greater attention paid to health at older ages, as well as the higher probability of having HTN and the greater experience of illness in older adults. In a study by Chow et al. [10] the prevalence of awareness of HTN varied from 46.9 to 57.4% in upper-middle-income countries and was also higher in older age groups. In the IHHP, the prevalence of awareness and treatment of HTN and controlled HTN in 2001 was 41.7, 35.4, and 9.8% and in 2008, 46.7, 40.0, and 14.0%, respectively [32]. Considering the similarity of the population of our study with that of the IHHP, it can be concluded that the status of awareness, treatment, and control of HTN improved during this time. The reasons for this may include improvements in the educational level of the population; the comprehensive and influential activities of national heath deputies, especially through national programs for the prevention and control of CVD; and improvements in insurance coverage and the availability of medical services in the past decade [33,34]. As a point of comparison, in 2 sequential surveys in Germany, the prevalence of awareness and treatment of HTN and controlled HTN was reported to be 69.4, 54.8, and 22.7% in 1998 and 82.3, 71.8, and 51.2% in 2011, respectively [35]. These comparisons show that the status of pharmacological treatment for HTN is good in Isfahan. We can attribute this to the exhaustive efforts of Isfahan’s national health institutes in training general physicians to prescribe medications for patients with HTN as soon as possible after diagnosis. Due to these regular training programs, both physicians and patients understood the importance of HTN and the role of pharmacological treatment for controlling this disease.

Although the status of awareness of HTN, treatment of HTN, and controlled HTN was satisfactory in our study, there was a considerable discrepancy between the proportion of treatment of HTN and controlled HTN among patients who were aware of having HTN (92.4 vs. 59.9%, respectively). This is in line with other studies. For example, in Germany [35] and Canada [36] these proportions were 71.8 vs. 51.2% and 79.0 vs. 64.6%, respectively. Nwankwo et al. [37] declared the Healthy People 2020 target goal on treatment of HTN not only to have been perfectly fulfilled, but to have exceeded the proposed goal (69.5%). However, there is still a substantial gap between the existing status and the proposed Healthy People 2020 target goal for HTN control (61.2%).

We can relate the high proportion of treatment of HTN in our study to the multiple efforts of the Isfahan Cardiovascular Research Institute (ICRI) since 2001. This institute has carried out comprehensive studies on cardiovascular risk factors [33,38]. The high prevalence of HTN as a traditional risk factor for CVD in these studies [39,40] has encouraged the ICRI to conduct numerous educational and operational activities focusing on the prevention and control of HTN through collaboration with the Isfahan national health institutes. One of the most important of these activities was the development of the first Iranian guideline on the prevention, evaluation, and management of HTN [41] by the Hypertension Research Center of the ICRI. The Isfahan national health institutes used this guideline in continuing medical education programs for general physicians. Thereby, general physicians, as core members of HTN therapy teams, have developed a deeper understanding of the importance of pharmacotherapy for HTN. These attempts were effective in achieving the good status of treatment in people who were aware of their HTN in our study. On the other hand, pharmacotherapy alone is not enough to control and manage HTN. Lifestyle improvements, including decreasing salt consumption and increasing dietary potassium via a diet rich in fruits and vegetables, managing body weight, engaging in more physical activity, and discontinuing alcohol drinking and tobacco smoking, along with pharmacological treatment, is necessary for control of HTN [42]. Since the relatively low control rate of HTN in our study was due to pitfalls in the behavioral patterns of patients, future initiatives should focus more on lifestyle modifications by engaging the patients in HTN management programs. Inviting them to participate in educational classes and providing educational materials in a plain and understandable language for the general public, preferably in multimedia formats, are some steps that will likely prove useful in this regard.

In general, the findings of this study showed that the prevalence of HTN was high, especially in men and in people over 40 as well as in postmenopausal women, and about one-third of the individuals with HTN were unaware of their condition. Another important finding of our study was that HTN was not controlled in nearly 40.1% of patients with HTN who were aware of their disease. Adopting a basic strategy for improving the timely diagnosis of this condition and its risk factors through community-wide screening programs is therefore necessary.

### Limitations and strengths

One of the limitations of this study, which is also present in all other epidemiological studies, is that the diagnosis of HTN was based on blood pressure measurements made during only one visit or on self-reports in the case of those who had HTN (with a diagnosis made by a physician or a member of the treatment team), and visiting the participants multiple times to make a definitive diagnosis of HTN was impractical for the research team. In addition, blood pressure in each person fluctuates across different hours of the day and night, but we determined whether an individual did or did not have HTN based on a single visit during the day.

The strengths of this study include using standard conditions to measure blood pressure, having a proper sample size, and selecting the subjects according to their gender and age ratios in the population.

### Implications for practice

Implementing programs to raise the public awareness of HTN is important, especially among young men and women, women near the age of menopause, and postmenopausal women. It is therefore necessary to inform these groups about the critical nature of HTN and ways to control its modifiable risk factors and to perform effective long-term health measures to help control HTN and its complications.

## Figures and Tables

**Table 1.** Demographic characteristics for all participants and by gender, Isfahan, Iran, 2016

Variables	Total	Women	Men	p-value
Age (yr)	39.53±15.74	39.45±15.56	39.61±15.90	0.81
BMI (kg/m^2^)	26.74±4.75	27.09±5.18	26.41±4.28	<0.001^1^
SBP (mmHg)	117.77±15.81	112.37±16.25	122.8±13.59	<0.001^1^
DBP (mmHg)	72.21±10.20	68.78±9.67	75.41±9.62	<0.001^1^
Years of education (yr)				<0.001^2^
0-5		249 (24.5)	147 (13.5)	
6-12		472 (46.4)	499 (45.8)	
>12		296 (29.1)	444 (40.7)	
Marital status				<0.001^2^
Single		148 (14.5)	288 (26.4)	
Married		788 (77.5)	789 (72.4)	
Separated/widow		81 (8.0)	13 (1.2)	
Occupation				<0.001^2^
Employed		110 (10.8)	752 (69.0)	
Unemployed		7 (0.7)	28 (2.5)	
Homemaker		769 (75.6)	0 (0.0)	
Student		113 (11.1)	125 (11.5)	
Retired		18 (1.8)	185 (17.0)	
Economic status				0.19
Poor		431 (42.4)	494 (45.3)	
Moderate		463 (45.5)	453 (41.6)	
Good		123 (12.1)	143 (13.1)	
Diabetes				0.10
Yes		99 (9.7)	88 (8.1)	
No		918 (90.3)	1,002 (91.9)	
Hyperlipidemia				0.06
Yes		177 (17.4)	162 (14.9)	
No		840 (82.6)	928 (85.1)	

Values are presented as mean±standard deviation or number (%). BMI, body mass index; SBP, systolic blood pressure; DBP, diastolic blood pressure.

1 p-values of the independent t-test.

2 p-values of the chi-square test.

**Table 2.** Comparison of selected risk factors for CVD in people with and without hypertension

Risk factors for CVD	Non-hypertensive (n=1,743)	Hypertensive (n= 364)	p-value
Age (yr)	35.84±13.28	57.21±14.51	<0.001^1^
BMI (kg/m^2^)	26.24±4.69	29.10±4.26	<0.001^1^
Gender			0.04^2^
Women	859 (84.5)	158 (15.5)	
Men	884 (81.1)	206 (18.9)	
Diabetes			<0.001^2^
Yes	89 (47.6)	98 (52.4)	
No	1,654 (86.1)	266 (13.9)	
Hyperlipidemia			<0.001^2^
Yes	194 (57.2)	145 (42.8)	
No	1,549 (87.6)	219 (12.4)	
Family history of hypertension			<0.001^2^
Yes	1,108 (80.0)	277 (20.0)	
No	635 (88.0)	87 (12.0)	
Current smoking			1.00^2^
Yes	158 (82.7)	33 (17.3)	
No	1,585 (82.7)	331 (17.3)	
Having a stressful life			0. 48^2^
Yes	1,351 (83.0)	276 (17.0)	
No	392 (81.7)	88 (18.3)	

CVD, cardiovascular disease; BMI, body mass index.

1 p-values of the independent t-test.

2 p-values of the chi-square test.

**Table 3.** Age-gender comparison of hypertension (HTN) prevalence, Isfahan, Iran, 2016

Age (yr)	Total	HTN			
		Total	Women	Men	p-value^1^
Total	2,107 (100.0)	364 (17.3)	158 (15.5)	206 (18.9)	0.01
18-29	718 (34.1)	26 (3.6)	1 (0.3)	25 (6.8)	<0.001
30-39	486 (23.1)	18 (3.7)	2 (0.9)	16 (6.3)	0.001
40-49	347 (16.5)	56 (16.1)	27 (15.8)	29 (16.5)	0.86
50-59	280 (13.3)	91 (32.5)	42 (31.1)	49 (33.8)	0.63
60-69	155 (7.4)	88 (56.8)	42 (57.5)	46 (56.1)	0.86
≥70	121 (5.7)	85 (70.2)	44 (78.6)	41 (63.1)	0.06
p-value^2^		<0.001	<0.001	<0.001	

Values are presented as number (%).

1 p-values of the the binomial test.

2 p-values of the chi-square test.

**Table 4.** Awareness, treatment, and control of HTN among people with a previous diagnosis of HTN, Isfahan, Iran, 2016

Age (yr)	Awareness		Pharmacological treatment		HTN control	
	Yes	No	Yes	No	Yes	No
Total	252 (69.2)	112 (30.8)	231 (92.4)	19 (7.6)	151 (59.9)	101 (40.1)
18-29	2 (7.7)	24 (92.3)	1 (50.0)	1 (50.0)	1 (50.0)	1 (50.0)
30-39	7 (38.9)	11 (61.1)	5 (71.4)	2 (28.6)	4 (57.1)	3 (42.9)
40-49	38 (67.9)	18 (32.1)	34 (89.5)	4 (10.5)	27 (71.1)	11 (28.9)
50-59	68 (74.7)	23 (25.3)	61 (91.0)	6 (9.0)	40 (58.8)	28 (41.2)
60-69	68 (77.3)	20 (22.7)	64 (94.1)	4 (5.9)	43 (63.2)	25 (36.8)
≥70	69 (81.2)	16 (18.8)	66 (97.1)	2 (2.9)	36 (52.2)	33 (47.8)
p-value^1^	<0.001		0.03		0.53	

Values are presented as number (%). HTN, hypertension.

1 p-values of the chi-square test.

**Table 5.** Results of logistic regression analysis evaluating the effects of various variables on the awareness of hypertension, Isfahan, Iran, 2016

Variables	OR (95% CI)	p-value
Age (yr)	1.10 (1.08, 1.12)	<0.001
Body mass index (kg/m^2^)	1.10 (1.06, 1.15)	<0.001
Gender (women)	3.55 (1.86, 6.78)	0.82
Diabetes (yes)	2.07 (1.36, 3.15)	0.001
Hyperlipidemia (yes)	2.05 (1.42, 2.95)	<0.001
Smoking (yes)	0.90 (0.50, 1.61)	0.72
Family history of hypertension (yes)	2.68 (1.85, 3.90)	<0.001

OR, odds ratio; CI, confidence interval.
